# *Angiostrongylus cantonensis* Meningitis and Myelitis, Texas, USA

**DOI:** 10.3201/eid2306.161683

**Published:** 2017-06

**Authors:** Roukaya Al Hammoud, Stacy L. Nayes, James R. Murphy, Gloria P. Heresi, Ian J. Butler, Norma Pérez

**Affiliations:** McGovern Medical School, University of Texas Health Science Center at Houston, Houston, Texas, USA

**Keywords:** meningitis, Texas, pediatric, *Angiostrongylus cantonensis*, parasitic diseases, helminths, zoonoses, parasites, United States, roundworms, nematodes, myelitis

## Abstract

Infection with *Angiostrongylus cantonensis* roundworms is endemic in Southeast Asia and the Pacific Basin. *A. cantonensis* meningitis and myelitis occurred in summer 2013 in a child with no history of travel outside of Texas, USA. Angiostrongyliasis is an emerging neurotropic helminthic disease in Texas and warrants increased awareness among healthcare providers.

In summer 2013, a previously healthy Caucasian 12-month-old girl was brought for treatment to a children’s hospital in Houston, Texas, USA, on the 11th day of illness (day 11), manifesting intermittent fever, lethargy, and emesis. She had been evaluated by a pediatrician on day 3 and diagnosed with presumed viral infection. She attended day care, had no history of sick contacts, and apart from dogs in the house, had no notable other exposures. 

At hospital admission, physical examination showed vital signs within reference ranges, mild distress, lethargy, and irritability with no focal deficits or signs of meningeal irritation. Blood test results showed leukocytosis (17,900 cells/mm^3^ with 20% eosinophils). Cerebrospinal fluid (CSF) examination showed 8 erythrocytes and 568 leukocytes/mm^3^ with 26% eosinophils. Results of bacterial cultures and PCR of CSF for herpes simplex virus and enterovirus were negative. She had no serologic evidence of acute infection with West Nile virus or HIV. Magnetic resonance imaging (MRI) of the brain showed normal results. She received ceftriaxone, vancomycin, and acyclovir from days 11 through 15 with no clinical improvement. 

On day 16, because the child had been exposed to dogs, she was empirically treated for presumed *Toxocara* infection with albendazole and prednisone for 5 days. Her clinical condition improved. However, 2 days after she completed the albendazole and prednisone regimen (day 23), fever and lethargy recurred, she was unable to bear weight, and CSF analysis showed increased eosinophilia (1,500 leukocytes/mm^3^ with 35% eosinophils). On day 23, MRI showed diffuse leptomeningeal enhancement in the brain and spinal cord, diffuse punctate areas of cortical infarction, and intramedullary enhancement (Figure). On the same day, she was again treated with albendazole and prednisone with good response. 

**Figure:**
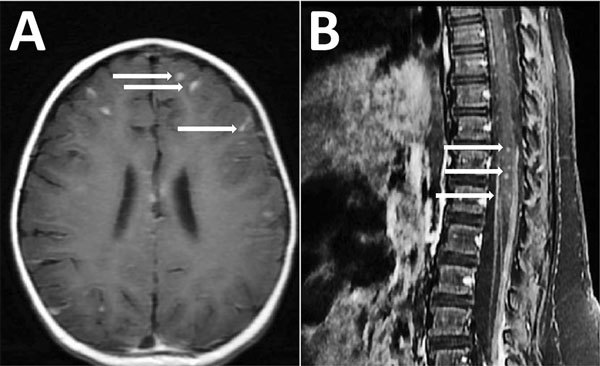
Magnetic resonance imaging (MRI) of the brain (A) and the spine (B) showing meningitis and myelitis in a 12-month-old girl with *Angiostrongylus cantonensis* infection, Houston, Texas, USA. A) Axial T1 post contrast sequences showing diffuse leptomeningeal enhancement (arrows). B) Sagittal T1 postcontrast sequences showing intramedullary enhancement in the thoracic and lumbar spinal cord T8–L5 with diffuse leptomeningeal enhancement (arrows).

Results of serologic testing performed at the hospital were negative for *Trichinella*, *Toxocara*, and *Schistosoma* spp. Serum and CSF samples were sent to the Centers for Diseases Control and Prevention (Atlanta, GA, USA); PCR results showed CSF was positive for *Angiostrongylus cantonensis*, and serum was positive for *Toxocara* *Strongyloides stercoralis* IgG. On day 31, we diagnosed *A. cantonensis* meningitis and myelitis with cross-reactions to *S. stercoralis* and *Toxocara* spp. The patient completed 2 weeks of albendazole therapy (from day 23 through day 37) and 3 months of prednisone therapy with slow tapering. The patient’s symptoms completely resolved by day 64. Lumbar punctures on days 35, 56, and 104 showed gradual return of CSF parameters to normal ranges. On day 56, results of an MRI of the brain were unremarkable. 

The case was reported to the local health department, which tested snails trapped close to the patient’s residence. No evidence of *A. cantonensis* roundworms was found.

In 1987, *A. cantonensis* roundworm was identified for the first time in North America in rodents. (*1*). Although a PCR-based assay did not detect *A. cantonensis* roundworm in samples of intermediate hosts (snails) in Houston, Texas, the parasite has been repeatedly identified in southeastern and Gulf states (Louisiana, Oklahoma, Mississippi, Florida) (*2**,**3*). In parallel, human infections have increased. The first reported human case of *A. cantonensis* meningitis in a nontraveler in the contiguous United States occurred in 1995 in a 10-year-old boy in New Orleans, Louisiana, who had eaten raw snails (*4*). Since the case we report, 2 additional cases in children were reported in Houston, in the spring of 2016 (*5*). 

The frequency of *A. cantonensis* infections in humans is likely underreported due to a combination of factors, including the frequent self-limited course of the infection, lack of awareness of the parasite, limited availability of diagnostic testing, and lack of national surveillance. Although the disease usually resolves spontaneously, case-fatality rates can reach 5% (*6*). Lack of clinical suspicion for angiostrongyliasis on the basis of signs and symptoms and delay in initiation of treatment can lead to neurologic deterioration, especially in young children, as demonstrated in our patient and another report (*7*). 

In conclusion, angiostrongyliasis is an emerging neurotropic helminthic disease in Texas. Increased awareness of *A. cantonensis* infection in humans is needed among healthcare providers.
